# Assessing coverage of essential maternal and child health interventions using health-facility data in Uganda

**DOI:** 10.1186/s12963-020-00236-x

**Published:** 2020-10-09

**Authors:** Elizabeth M. Simmons, Kavita Singh, Jamiru Mpiima, Manish Kumar, William Weiss

**Affiliations:** 1 Department of Maternal and Child Health, Gillings School of Global Public Health, University of North Carolina at Chapel Hill, Chapel Hill, NC USA; 2 D4I Project, Carolina Population Center, Chapel Hill, NC USA; 3 Makerere University, Kampala, Uganda; 4 Department of International Health, Bloomberg School of Public Health, Johns Hopkins University, Baltimore, MD USA; 5 Public Health Institute, Oakland, CA USA

**Keywords:** Data adjustments, Data quality, Health information systems, Maternal and child health, Uganda

## Abstract

**Background:**

Nationally representative household surveys are the gold standard for tracking progress in coverage of life-saving maternal and child interventions, but often do not provide timely information on coverage at the local and health facility level. Electronic routine health information system (RHIS) data could help provide this information, but there are currently concerns about data quality. This analysis seeks to improve the usability of and confidence in electronic RHIS data by using adjustments to calculate more accurate numerators and denominators for essential interventions.

**Methods:**

Data from three sources (Ugandan Demographic and Health (UDHS) survey, electronic RHIS, and census) were used to provide estimates of essential maternal (> 4 antenatal care visits (ANC), skilled delivery, and postnatal care visit (PNC)) and child health interventions (diphtheria, pertussis, tetanus, and hepatitis B and *Haemophilus influenzae* type b and polio vaccination series, measles vaccination, and vitamin A). Electronic RHIS data was checked for quality and both numerators and denominators were adjusted to improve accuracy. Estimates were compared between the three sources.

**Results:**

Estimates of maternal health interventions from adjusted electronic RHIS data were lower than those of the UDHS, while child intervention estimates were typically higher. Adjustment of electronic RHIS data generally improved accuracy compared with no adjustment. There was considerable agreement between estimates from adjusted, electronic RHIS data, and UDHS for skilled delivery and first dose of childhood vaccination series, but lesser agreement for ANC visits and second and third doses of childhood vaccinations.

**Conclusions:**

Nationally representative household surveys will likely continue being the gold standard of coverage estimates of maternal and child health interventions, but this analysis shows that current approaches to adjusting health facility estimate works better for some indications than others. Further efforts to improve accuracy of estimates from RHIS sources are needed.

## Background

Nationally representative household surveys are typically considered the gold standard in low- and middle-income countries for tracking progress in the coverage of life-saving maternal, newborn, and child health interventions given concerns about the data quality of countries’ routine health information systems (RHIS) [1]. Of note, however, is that these surveys are conducted on a 3 to 5-year cycle and provide estimates at the national and subnational levels, but often neither at the lowest operational nor at the health-facility catchment level [2]. As a result, population-based surveys are not used for real-time (or near real-time) monitoring of health care utilization, service delivery, or health system functioning, especially at the local level [3]. Electronic RHIS data, compiled from health facility (and in cases community based) data, are becoming increasingly important as government health systems decentralize and there is a need for officials at the district and facility levels to have more frequently available data to make financial and managerial decisions and be held accountable for these decisions [4].

The District Health Information Software Version 2 (DHIS2) is a free and open-source software platform used to develop electronic RHIS in over 60 countries in Africa and Asia [5]. The implementation of electronic RHIS using the DHIS2 has led to improvements in both completeness and timeliness of data reporting of health facility data [6, 7]. Despite the benefits of electronic RHIS, there are still problems of completeness and timeliness of reporting, consistency over time, and consistency between facility data and routine health survey data [8, 9]. In order to improve usability of and decision-making around electronic RHIS data, there is a need to improve the estimation of target populations with this data to provide more accurate denominators [10], as well as improve the estimation of intervention coverage to improve numerator estimates.

This analysis will add to the methodology set forth by Maina et al. [1] to improve the usability of health facility data by calculating more accurate numerators and denominators for essential maternal and child interventions in Uganda. We hypothesize that creating more confidence in the accuracy of electronic RHIS data will lead to more use of these data as well as better decision-making around the use of these data. In this analysis, coverage estimates of selected interventions were calculated using unadjusted and adjusted denominators from electronic RHIS data (using the DHIS2 software), and comparisons were made to both nationally representative survey estimates and estimates calculated using census-based population estimates as the denominator.

## Methods

### Study setting

The DHIS2 was adopted in Uganda at the national level in January 2011 in order to further develop its national, electronic RHIS [11], leading to many improvements in data reporting of health facilities [7]. Uganda has a decentralized health system, with the public, private sectors and donors playing major roles. Its health system is organized in a hierarchical fashion with three levels of health centers, at the village (village health teams), parish (health center II), and subcounty levels (health center III) feeding into a health facility (health center IV) at the subdistrict level. These health facilities then feed into a district-level referral hospital. Each region has an overarching referral hospital as well, with the National Referral Hospitals in Kampala.

### Data sources

The necessary data and statistics for this analysis were obtained from three data sources. The Demographic and Health Surveys (DHS) program has collected and analyzed nationally representative data on population and health, including maternal and child health, in over 90 countries since 1984 [12]. The most recent Uganda Demographic and Health Survey (UDHS) in 2015-2016 provided estimates of coverage of maternal and child interventions at the national and subnational levels in Uganda. This study used UDHS estimates of coverage for each subregion as the gold standard. Data from the DHIS2 software were used to assess estimates of health facility coverage of these interventions at the district level in both 2015 and 2016. We will hereon refer to electronic RHIS data in Uganda as DHIS2 data, as the DHIS2 software was the source of the data. The DHIS2 estimates were aggregated by subregion and in order to provide a comparison with the UDHS data. Then, these data were adjusted to improve the quality of the numerators and denominators. In addition, Uganda has undertaken five population censuses since gaining independence, the most recent of these being the 2014 National Population and Housing Census [13]. These data were used to provide a second, population-based denominator for comparison. Census-based denominators were combined with numerators from the DHIS2 data to calculate census-adjusted estimates of coverage for child interventions for each subregion. We were unable to calculate census-adjusted coverage estimates from DHIS2 for maternal interventions as census data did not include information on the appropriate denominator: expected pregnancies.

For maternal interventions, UDHS-adjusted coverage estimates were calculated by subregion for the following indicators: (i) at least one antenatal care (ANC) visit; (ii) 4 or more ANC visits; (iii) a postnatal care (PNC) visit within 6 days of delivery; and (iv) skilled attendance at birth. Four or more ANC visits were chosen as the focused ANC model of ANC care, which included four visits, was the standard of care at the time of data collection for our analysis [14]. For child health interventions, UDHS- and census-adjusted estimates were calculated by subregion for the following indicators: (i) Bacille Calmette-Guerin (BCG) vaccination; (ii) the three-part diphtheria, pertussis, tetanus, and hepatitis B and *Haemophilus influenzae* type b (DPT-HepB-Hib) vaccination series; (iii) the three-part polio vaccination series; (iv) measles vaccination; and, (v) receipt of vitamin A. Vitamin A coverage estimates were restricted to children between 6 and 11 months for the UDHS and census data. Children under the age of 1 were used as the age group for DHIS2 for vaccinations, and for vitamin A as it were not possible to disaggregate vitamin A data down to the same level using the DHIS2 software.

### Data quality checks

DHIS2 data were checked for quality by looking for district-level outliers in the estimates for selected interventions, and for completeness of facility reporting at the subregion level. The percentage of districts with at least one monthly outlier was calculated for each maternal and child intervention for 2015 and 2016 separately. For each district, the reported monthly number of women or children receiving an intervention was classified as an outlier if its value was more than two standard deviations away from the annual mean of that district. Table 1 shows the percentage of districts with at least one monthly outlier and the number of districts with greater than 1 monthly outlier in 2015 and 2016. For all included interventions, except vitamin A, the percentage of districts with at least one monthly outlier is between 35.9 and 48.4 percent. For vitamin A, over 90% of districts had at least one monthly outlier in 2016.

**Table 1:** Percentage of districts with at least one monthly outlier^a^ and number of districts with > 1 monthly outlier in DHIS2 estimates for the number of women/children receiving each intervention in 2015 and 2016

Intervention	% of districts with **>** 1 monthly outlier in 2015	% of districts with **>** 1 monthly outlier in 2016	# districts with > 1 outlier in 2015	# districts with > 1 outlier in 2016
**Maternal interventions**				
At least 1 ANC visit	37.5	41.4	1	0
≥ 4 ANC visits	43.0	42.2	0	0
Skilled delivery	47.7	45.3	1	2
PNC within 6 days of delivery^b^		48.4		0
**Child interventions**				
BCG^2^		35.9		0
DPT-Hib-HepB 1^b^		44.5		0
DPT-Hib-HepB 2^b^		46.9		0
DPT-Hib-HepB 3^b^		39.1		1
Polio 1^b^		44.5		1
Polio 2^b^		44.5		1
Polio 3^b^		45.3		0
Measles^b^		45.3		1
Vitamin A^b^		93.0		1

^a^For each district, the annual mean and standard deviations were calculated based on monthly numbers of women/children receiving each intervention. A monthly report was identified as an outlier if it was more than two standard deviations away from the annual mean.

^b^Incomplete data for 2015

Reporting rates were calculated for each subregion by dividing the number of facilities submitting monthly reports for 2015-2016 by the total number of expected reports, grouping maternal and child interventions separately. Reporting rates at the subregion level varied from 51 to 95% for maternal interventions and from 54 to 95% for child interventions. Three subregions had reporting rates less than 80% for maternal interventions and two had reporting rates less than 80% for child interventions. Reporting rates are included in Supplementary Table 1, along with other factors used for adjusting DHIS2 numbers.

### DHIS2 numerator adjustments

Numerators were first obtained from unadjusted DHIS2 data for the number of women and children reported to have received the individual interventions. The raw numbers for maternal interventions were adjusted for private sector use according to the UDHS as the DHIS2 does not capture all services provided in the private sector. Attending a PNC visit within 6 days of delivery was additionally adjusted for private sector and home use, as over 50% of PNC visits within 6 days of delivery were done either at home or in private facilities in most subregions [15]. Supplementary Table 1 shows adjustment data by subregion and data source. In addition, numerators for skilled delivery and receiving PNC within 6 days were adjusted for twins, based on the twinning rate of Uganda which is 15.4 per 1000 births [16]. The numerators for child interventions were not adjusted for immunizations occurring in the private sector as this information was not available in the UDHS.

### DHIS2 denominator adjustments

#### DHIS2 denominator choices for maternal and child interventions

Denominators from the UDHS and census were left unadjusted. For maternal interventions, the number of women who attended at least one ANC visit was used as the denominator for the three included interventions. The indicator for having at least one ANC visit during pregnancy is high and consistent in Uganda with between 93.6 and 99.8% of pregnant women in each subregion attending their first ANC visit [15], thus providing the best estimate at the appropriate denominator for maternal interventions: expected pregnancies. For child interventions, the number of children receiving BCG is also high across Uganda as between 92.5 and 99.3% of all children receive it and was used as the denominator for the selected interventions [15]. For coverage estimates of the DPT-HepB-Hib and polio vaccination series, we also used the number of children who received the first vaccination in the series as the denominator of the second and third vaccinations, for comparison.

#### Adjustment for incomplete reporting

DHIS2-based denominators were first adjusted for incomplete reporting with the following equation from Maina et al [1]:

$$ {N}_{adjusted}={N}_{reported}\ast \left(\frac{1}{c}-1\right)\ast k $$

where *c* is reporting completeness and *k* is the adjustment factor that represents the expected level of service at the non-reporting facilities. The reporting completeness variables for maternal and child interventions, represented in the above equation by *c*, are included in Supplementary Table 1. If missing reports are an indication that no services were provided at these facilities during the reporting period then *k* = 0, but if it is possible that services were provided, but at a lower level than those facilities with complete reports then *k* is between 0 and 1. As we were uncertain what the appropriate *k* value was for the Ugandan context, we adjusted the denominators by five *k* values: 0, 0.25, 0.5, 0.75, and 1.

#### Adjustment for non-use of services

Next, DHIS2-based denominators were adjusted for non-use of the services. The proportion of women who did not attend at least one ANC visit and children who did not receive BCG, DPT-HebB-Hib1, and Polio 1 were calculated by subregion from the UDHS. The DHIS2-based denominator for each subregion was then inflated by these values (Supplementary Table 1).

#### Adjustment for stillbirths

Finally, the DHIS2-denominators for maternal interventions were further adjusted to account for stillbirths. Stillbirths cause a change in denominator between the first ANC visit and the 4th ANC visit, delivery, or PNC visit. The stillbirth rate in Uganda is 21 per 1000 [17]. About half of these stillbirths occur in the antepartum period and half during labor and delivery [18]. The denominator for at least 4 ANC visits was deflated by half the stillbirth rate (or 0.0105) and the denominator for skilled delivery and PNC visit was deflated by the full stillbirth rate (0.021) [17].

### Calculation of coverage estimates from adjusted-DHIS2 data

Coverage estimates of (a) 4 or more ANC visits, (b) PNC visit within 6 days of delivery, and (c) skilled delivery were calculated by dividing the adjusted DHIS2 numerators by the adjusted-DHIS2 first ANC visit denominators for both 2015 and 2016. Coverage estimates of the (a) DPT-HepB-Hib vaccination series, (b) polio vaccination series, (c) measles vaccination, and (d) receipt of vitamin A were calculated by dividing the DHIS2 numerators (non-adjusted) by the adjusted-DHIS2 BCG denominator within each subregion in 2016. Coverage estimates for the second and third vaccinations of the DPT-HepB-Hib and polio vaccinations series were also calculated by dividing the DHIS2 numerators (non-adjusted) by the adjusted DPT-HepB-Hib1 and Polio1 denominators, respectively. DHIS2 data for PNC within 6 days of delivery and all child interventions were incomplete in 2015 and therefore excluded from the 2015 analysis.

The adjusted maternal intervention coverage estimates from the DHIS2 were then compared with the UDHS and the unadjusted-DHIS2 estimates. The adjusted child intervention coverage estimates from the DHIS2 were compared with the UDHS, the census, and the unadjusted-DHIS2 numbers. The percent difference between the UDHS and DHIS coverage estimates (unadjusted and adjusted) were compared by subregion. The percent of subregions for which there was a difference of less than 10% and less than 20% between the DHIS2 coverage estimates and UDHS was calculated.

## Results

Coverage estimates for four or more ANC visits and skilled attendance at birth from the UDHS and the unadjusted and adjusted DHIS2 are shown in Table 2. All adjusted-DHIS2 estimates for maternal interventions are with *k* = 0 as this value of *k* consistently produced the most accurate estimates (sensitivity analysis for different values of *k is* not shown). Unadjusted-DHIS2 estimates are significantly lower than those of the UDHS with adjusted-DHIS2 estimates closer to the UDHS in both 2015 and 2016. The same trend is seen in coverage estimates of skilled delivery, with two exceptions. First, in two subregions both the unadjusted and adjusted DHIS2 estimates in 2015 are significantly higher than those DHIS2 estimates of 2016 (Kampala and South Central). Second, in the Kigezi subregion, the unadjusted-DHIS2 estimates are more similar to the UDHS than those of the adjusted DHIS2.

**Table 2:** Coverage estimates of at least 4 ANC visits and skilled delivery from UDHS and unadjusted- and adjusted-DHIS2 numbers by subregion for 2015 and 2016

Intervention		ANC4+					Skilled delivery				
Source		UDHS	DHIS				UDHS	DHIS			
Adjustment		No	No		Yes^**a**^		No	No		Yes^**a**^	
Year		2015-2016	2015	2016	2015	2016	2015-2016	2015	2016	2015	2016
**Subregions**	Acholi	60.1%	48.1%	44.5%	54.0%	50.0%	84.1%	72.4%	71.5%	85.1%	84.0%
	Ankole	67.5%	50.8%	50.0%	57.6%	56.7%	70.6%	62.9%	66.0%	73.8%	77.5%
	Bugisu	48.8%	33.2%	33.6%	33.7%	34.1%	56.2%	47.9%	50.6%	50.4%	53.2%
	Bukedi	55.3%	40.1%	36.7%	41.8%	38.2%	66.0%	61.0%	63.6%	64.7%	67.4%
	Bunyoro	46.5%	32.3%	30.9%	33.2%	31.7%	56.9%	43.9%	47.5%	44.9%	48.6%
	Busoga	65.0%	32.5%	35.2%	34.5%	37.4%	76.5%	44.7%	47.7%	54.7%	58.3%
	Kampala	69.0%	36.1%	32.7%	46.6%	42.2%	94.3%	137.5%	58.9%	186.6%	79.9%
	Karamoja	65.7%	43.2%	46.5%	47.9%	51.5%	71.2%	63.3%	63.2%	69.0%	68.9%
	Kigezi	60.5%	48.2%	46.9%	54.7%	53.3%	69.7%	69.6%	73.4%	80.5%	85.0%
	Lango	57.0%	42.1%	40.6%	45.6%	43.9%	66.3%	57.1%	60.4%	63.2%	66.8%
	North Central	58.7%	35.8%	35.6%	39.4%	39.2%	74.7%	51.1%	53.3%	61.0%	63.7%
	South Central	66.5%	35.4%	34.8%	43.5%	42.7%	81.1%	90.8%	51.9%	119.6%	68.3%
	Teso	53.3%	32.5%	33.2%	33.9%	34.7%	73.9%	58.3%	59.9%	61.5%	63.1%
	Tooro	63.4%	47.1%	51.3%	53.3%	58.2%	73.6%	54.8%	59.6%	63.6%	69.1%
	West Nile	65.1%	45.8%	47.4%	52.1%	53.8%	78.2%	68.1%	73.4%	75.9%	81.7%

^a^Denominator adjustment using *k* = 0

Coverage estimates of having a PNC visit within 6 days of delivery from the UDHS, and the unadjusted and adjusted DHIS2 (with numerators adjusted for both private sector use only and private and home sector use together) are shown in Table 3. Adjusted-DHIS2 estimates are also all produced with a *k* value of 0 as this *k* value produced the most comparable estimates to those of the UDHS (sensitivity analysis of different *k* values not shown). All estimates from the DHIS2 are significantly lower than those of the UDHS.

**Table 3:** Coverage estimates of PNC visit within 6 days of delivery from UDHS, unadjusted- and adjusted-DHIS2 numbers by subregion in 2015-2016

Intervention		PNC within 6 days of delivery			
Source		UDHS	DHIS2		
Denominator adjustment		No	No	Yes^**a**^	
Numerator adjustment		N/A	N/A	Private use	Private + home Use
Year		2015-2016	2016	2016	2016
**Subregions**	Acholi	53.8%	9.2%	10.2%	14.0%
	Ankole	42.6%	8.2%	9.8%	13.1%
	Bugisu	56.4%	5.3%	5.3%	8.8%
	Bukedi	59.9%	3.9%	4.1%	6.4%
	Bunyoro	39.1%	4.1%	4.1%	6.9%
	Busoga	43.6%	6.3%	6.6%	11.0%
	Kampala	77.6%	6.4%	9.2%	10.4%
	Karamoja	86.7%	16.2%	16.9%	26.2%
	Kigezi	48.3%	4.1%	4.5%	5.7%
	Lango	55.5%	3.4%	3.5%	5.7%
	North Central	57.8%	3.8%	4.6%	6.1%
	South Central	57.1%	3.9%	5.2%	6.5%
	Teso	66.0%	14.0%	14.8%	23.3%
	Tooro	44.7%	8.7%	10.0%	13.2%
	West Nile	60.9%	9.2%	10.1%	14.9%

^a^Denominator adjustment using *k* = 0

**Fig. 1:**
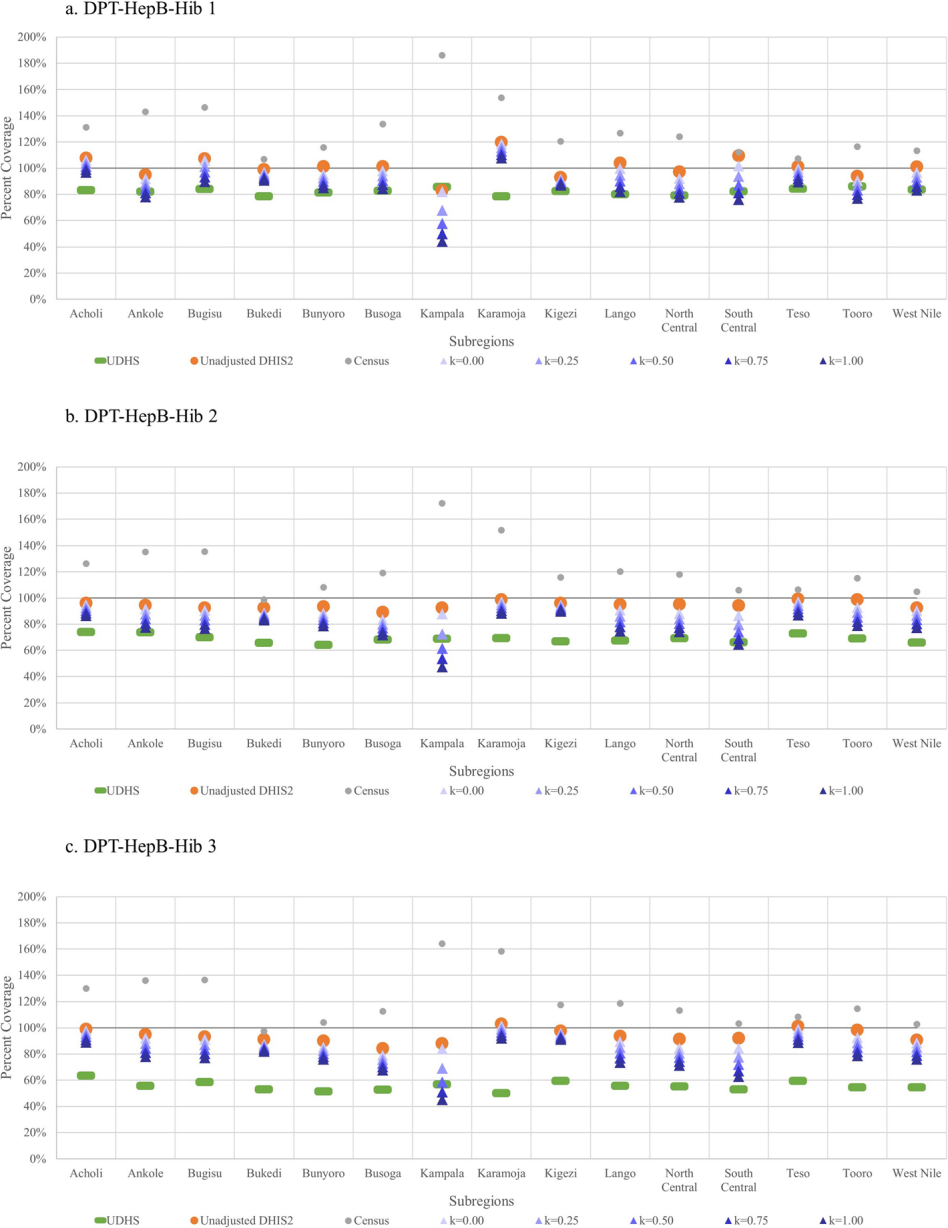
Coverage estimates of the DPT-HepB-Hib vaccination series from UDHS, census, unadjusted- and adjusted-DHIS2 data

**Fig. 2:**
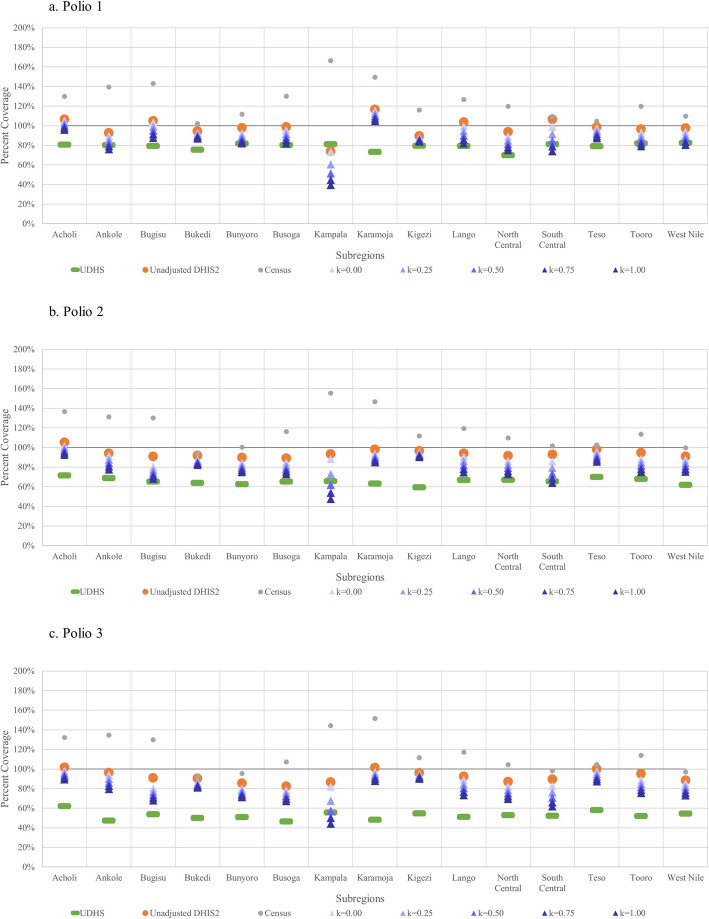
Coverage estimates of the polio vaccination series from UDHS, census, unadjusted- and adjusted-DHIS2 data

Figure 1 shows coverage estimates of the DPT-HepB-Hib vaccination series by subregion and the following sources: UDHS, census, unadjusted DHIS2, and adjusted DHIS2 with different *k* values. Unlike maternal interventions, all *k* values are shown for child interventions as the *k* value that produced the most accurate estimate differed by subregion. Census estimates for vaccination coverage are consistently above 100%. In general, adjusted-DHIS2 estimates are closer to that of UDHS than unadjusted estimates and higher than those of the UDHS. Similar results for the polio vaccination series are seen in Fig. 2.

**Fig. 3:**
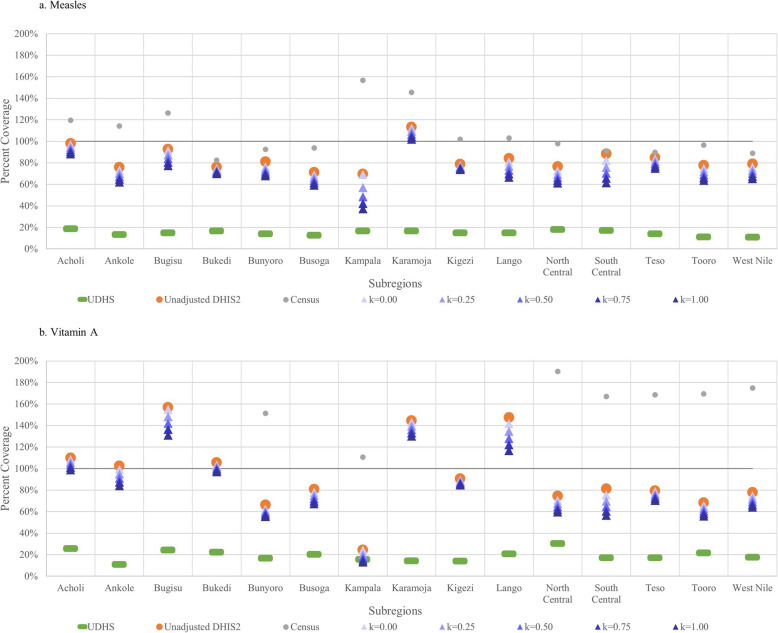
Coverage estimates of the measles and vitamin A from UDHS, census, unadjusted- and adjusted-DHIS2 data

Figure 3 shows estimates of coverage from UDHS, census, and unadjusted- and adjusted-DHIS2 data of measles and vitamin A by subregion. Unlike the DPT-HepB-Hib and polio vaccination series coverage estimates, the adjusted DHIS2 estimates of measles and vitamin A are significantly higher than those of UDHS in all subregions, but Kampala.

Table 4 shows the percent of subregions for which there was a difference of 10% or less and 20% or less between coverage estimates from the DHIS2 (adjusted and unadjusted) as compared to the UDHS. Denominators for the maternal interventions are adjusted with *k* = 0, and the numerators are adjusted for private sector use only. Child intervention denominators are adjusted with *k* = 1 and the vaccination series denominators are based on the first vaccination in the series. For having at least 4 ANC visits, there is moderate agreement between UDHS and adjusted-DHIS2 estimates with 7% of subregions having < 10% difference and 33% of subregions having ≤ 20% in 2015 and 2016. There is no agreement between the UDHS and adjusted-DHIS2 estimates for having a PNC visit within 6 days. Estimates of skilled delivery from UDHS and the adjusted-DHIS2 data compare well, with 40 and 53% of subregions having a ≤ 10% difference and 73 and 87% of subregions having ≤ 20% difference in 2015 and 2016, respectively. For child interventions, there is also frequent agreement between UDHS and adjusted-DHIS2 estimates for first DPT-HepB-Hib and polio vaccinations with 87% of subregions having ≤ 20% difference for both interventions. There is moderate agreement between UDHS and adjusted-DHIS2 estimates for the second in the vaccination series and almost no agreement in coverage estimates for the third dose. There is no agreement in the measles and vitamin A estimates. Across almost all interventions, adjusting the DHIS2 numbers improves agreement.

**Table 4:** Percent difference in maternal and child interventions from UDHS to unadjusted- and adjusted-DHIS2 estimates

	Percent of subregions with a ≤ 10% difference in UDHS and DHIS2 estimates		Percent of subregions with a ≤ 20% difference in UDHS and DHIS2 estimates	
Intervention	No. adjustment	Adjustment	No. adjustment	Adjustment
**Maternal interventions**^a^				
ANC 4+ visits (2015)	0	7	0	33
ANC 4+ visits (2016)	0	7	7	33
PNC within 6 days (2016)^b^	0	0	0	0
Skilled delivery (2015)	13	40	6	73
Skilled delivery (2016)	40	53	73	87
**Child interventions**^c^				
DPT-HepB-Hib 1^b^	13	67	27	87
DPT-HepB-Hib 2^b,d^	0	33	0	67
DPT-HepB-Hib 3^b,d^	0	0	0	7
Polio 1^b^	7	60	40	87
Polio 2^b,e^	0	20	0	53
Polio 3^b,e^	0	0	0	7
Measles^b^	0	0	0	0
Vitamin A^b^	0	0	0	7

^a^Maternal intervention denominators are adjusted using *k* = 0 and numerators are adjusted for private sector use only

^b^Complete data was not available for 2015 from the DHIS2 for these interventions

^c^Child intervention denominators are adjusted using *k* = 1

^d^DPT-HepB-Hib 2 and 3 denominators are based on number of children who received DPT-HepB-Hib 1

^e^Polio 2 and 3 denominators are based on number of children who received Polio 1

## Discussion

In this study, we compared coverage estimates of essential maternal and child interventions from a nationally representative household survey to those derived from unadjusted- and adjusted-health facility service statistics. We found that there was considerable agreement for the following interventions: skilled attendance at birth; and the first doses of DPT-HepB-Hib and polio vaccinations (polio birth dose not analyzed). There was moderate agreement between adjusted-DHIS2 and UDHS coverage estimates for at least four ANC visits and the second vaccinations in each series. There was no agreement between the adjusted-DHIS2 and the UDHS coverage estimates for the third vaccination dose in each series, for measles vaccination, and for vitamin A. Coverage estimates for child interventions produced using the census also showed no agreement with estimates derived from DHIS2 (adjusted and non-adjusted), and the census-derived estimates were often much higher than 100% (which is usually not possible without unusual levels of visitors coming from other catchment areas for these services).

Other studies have used a similar methodology to improve the accuracy of coverage estimates derived from routinely collected health facility data for key maternal and child health indicators. Similar to our findings, Maina et al. found that similarly adjusted facility-based data in Kenya (also from Kenya’s DHIS2) produced coverage estimates for health facility delivery that were similar to the estimates from the Kenya DHS. However, the coverage estimates for at least four ANC visits was lower for the adjusted DHIS2 as compared to the Kenya DHS [1]. Unlike our analysis, Maina et al. calculated adjusted-DHIS2 coverage estimates for the first and third doses in the DPT-HepB-Hib vaccination series that were similar to those of the Kenya DHS [1]. Another study found that health facility data were more accurate for estimating contact indicators, such as ANC, skilled delivery, and PNC, than for estimating health facility indicators that involve the provision of commodities (such as vaccines and vitamin A) [8].

A number of factors could explain the underreporting of maternal interventions in health facility data compared with UDHS data. First, women may seek care from health facilities outside of their subregion [10] or outside of the health facility completely, reducing the accuracy of coverage estimates of maternal and child interventions derived from health facility data alone. Women who travel outside their subregion to receive care could not only explain the lower coverage estimates of health facility data in most subregions but also explain the above 100% coverage reported in health facility data in Kampala. It is also possible that as patients are referred from one facility to another, their records may not follow them, leading to an underreporting of certain services, such as ANC visits. Second, the authors of the Maina et al. analysis raised the idea that the DHS may not be a gold standard for estimates of the number of ANC visits (with possible over-reporting based on respondent recall) and that the truth may be somewhere in between, or perhaps even closer to the adjusted-DHIS2 estimates [1]. Finally, some services might be provided in the private sector, which does not completely report to the DHIS2. The extent to which the private does report to the DHIS2 needs further examination.

There are also possible explanations for the overreporting of child interventions in health facility data compared to that of the UDHS. First, we may not be sufficiently adjusting the denominator for child interventions in order to capture the true population who would be receiving these interventions. Second, there could be pressure to report higher coverage of vaccinations through facility reports than actual vaccinations provided. A study in Uganda found that poor record keeping led to inaccurate immunization records in health facilities [19]. Finally, there could be recall bias in estimates produced by the UDHS making it possible that the true estimate is somewhere between that of the UDHS and health facility data [1]. It is also possible to explain the variation in estimates between subregions. Stockouts could have affected the ability of facilities to provide immunizations and it is unclear the extent to which women and children traveled in order to gain access to essential vaccinations. Stockouts could help explain the variation in vaccination coverage by subregion seen in DHIS2 estimates. For example, a study of Hoima District found stockouts to be a barrier in the provision of immunization services [20].

This study has several strengths. To our knowledge, it is the first study in Uganda to use this methodology to improve coverage estimates of essential maternal and child health interventions using health facility data. The DHIS2 values used to calculate the denominators for DHIS2 estimates (i.e., at least one ANC visit, receipt of BCG vaccination) had consistently high UDHS coverage levels (90% or higher) across Uganda helping to produce an alternative, population-based estimate of the size of key populations in each subregion.

This study also has several limitations. Frequent changes to administrative boundaries could complicate population projections and therefore the denominators of the census and DHIS2 estimates [10]. However, we made our best attempt to define the subregions in a consistent fashion across the three data sources to circumvent this issue by matching newer districts in the DHIS2 and census with subregions in the UDHS in order to avoid double-counting districts toward the denominators. We were also unable to adjust DHIS2 denominators for the migration effect, as these data were unavailable. However, we aggregated the DHIS2 data used in this analysis to the subregional level which should compensate for inter-facility catchment area movement within each subregion. Denominators used for coverage estimates of child health interventions from the DHIS2 were not adjusted for private sector use as this information was not available in the UDHS. The ideal denominator for PNC visits should be expected deliveries, but we were unable to estimate expected deliveries by adjusting expected pregnancies by the rate of miscarriages or abortions.

## Conclusion

Nationally representative household surveys will likely continue being the gold standard for population-based coverage estimates of maternal and child health interventions. However, there is increasing demand for more frequent estimates and for estimates that represent smaller areas than national household surveys like the DHS provide. Facility data currently provide more frequent estimates of these interventions at both a national and local levels but the quality of these estimates is suspect, hindering confidence to use these estimates for making appropriate decisions. This and other analyses show that current approaches to adjusting facility-based coverage estimates using population-based sources work better for some indicators than others, and that accuracy of these adjustments vary by country and data source (e.g., survey vs census). Further efforts to improve the accuracy of coverage estimates based on routine health facility data are needed, as well as a better understanding of the conditions when these improvement methods are sufficient and for how long these estimates would be valid.

## Supplementary information

**Additional file 1.** : Supplementary Table 1. Adjustment factors for DHIS2 numerators and denominators.

## Data Availability

The datasets used and/or analyzed during the current study are available from the corresponding author on reasonable request. These data are also publicly accessible.

## Abbreviations

ANC: Antenatal care
BCG: Bacille Calmette-Guerin
DHIS2: District Health Information Software Version 2
DHS: Demographic and Health Surveys
DPT: Diphtheria, pertussis, tetanus
HepB: Hepatitis B
Hib: *Haemophilus influenzae* type b
PNC: Postnatal care
RHIS: Routine Health Information System
UDHS: Uganda Demographic and Health Survey
