# Supplementary material for: Assessing coverage of essential maternal and child health interventions using health-facility data in Uganda
Source: Popul Health Metr. 2020 Oct 9;18:26. doi: 10.1186/s12963-020-00236-x (PMC7547522; doi:10.1186/s12963-020-00236-x)
Supplement: Supplementary file 1 — Additional file 1. : Supplementary Table 1. Adjustment factors for DHIS2 numerators and denominators. [file 12963_2020_236_MOESM1_ESM.docx]

Supplementary Table 1. Adjustment factors for DHIS2 numerators and denominators

| **Adjustment Factor** | **Non-use of ANC1** | **Non-use of BCG** | **Non-use of DPT-HepB-Hib1** | **Non-use of Polio1** | **Reporting rates for maternal interventions** | **Reporting rates for child interventions** | **Stillbirth rate** | **1/2 Stillbirth rate** | **Private Sector Use - ANC** | **Private Sector Use - Skilled Delivery** | **Private Sector Use - PNC** | **Private Sector and Home Use - PNC** | **Twinning rate** |
| --- | --- | --- | --- | --- | --- | --- | --- | --- | --- | --- | --- | --- | --- |
| **Data Source** | **UDHS**^14^ | **UDHS**^14^ | **UDHS**^14^ | **UDHS**^14^ | **DHIS2** | **DHIS2** | **UNICEF**^16^ | **UNICEF**^16^ | **UDHS**^14^ | **UDHS**^14^ | **UDHS**^14^ | **UDHS**^14^ | **Smits & Monden, 2011**^15^ |
| **Acholi** | 0.0060 | 0.0130 | 0.0130 | 0.0350 | 0.8881 | 0.9120 | 0.0210 | 0.0105 | 0.1190 | 0.1570 | 0.0938 | 0.5000 | 0.0154 |
| **Ankole** | 0.0300 | 0.0330 | 0.0310 | 0.0260 | 0.8224 | 0.8478 | 0.0210 | 0.0105 | 0.1557 | 0.1840 | 0.1951 | 0.6098 | 0.0154 |
| **Bugisu** | 0.0220 | 0.0130 | 0.0210 | 0.1290 | 0.8320 | 0.8465 | 0.0210 | 0.0105 | 0.0277 | 0.0520 | 0.0147 | 0.6691 | 0.0154 |
| **Bukedi** | 0.0200 | 0.0220 | 0.0440 | 0.0480 | 0.9177 | 0.9392 | 0.0210 | 0.0105 | 0.0533 | 0.0590 | 0.0410 | 0.6311 | 0.0154 |
| **Bunyoro** | 0.0640 | 0.0620 | 0.0560 | 0.0670 | 0.8851 | 0.8906 | 0.0210 | 0.0105 | 0.0817 | 0.0640 | 0.0635 | 0.7619 | 0.0154 |
| **Busoga** | 0.0160 | 0.0330 | 0.0690 | 0.0510 | 0.8567 | 0.8604 | 0.0210 | 0.0105 | 0.0682 | 0.2160 | 0.0319 | 0.7234 | 0.0154 |
| **Kampala** | 0.0210 | 0.0070 | 0.0520 | 0.0560 | 0.5131 | 0.5394 | 0.0210 | 0.0105 | 0.3053 | 0.3570 | 0.4444 | 0.6333 | 0.0154 |
| **Karamoja** | 0.0180 | 0.0110 | 0.0150 | 0.0470 | 0.8976 | 0.9091 | 0.0210 | 0.0105 | 0.1170 | 0.0860 | 0.0404 | 0.6162 | 0.0154 |
| **Kigezi** | 0.0020 | 0.0170 | 0.0170 | 0.0150 | 0.9475 | 0.9514 | 0.0210 | 0.0105 | 0.1263 | 0.1360 | 0.0652 | 0.3478 | 0.0154 |
| **Lango** | 0.0140 | 0.0400 | 0.0450 | 0.0370 | 0.7837 | 0.8228 | 0.0210 | 0.0105 | 0.0852 | 0.0980 | 0.0435 | 0.6783 | 0.0154 |
| **N. Central** | 0.0120 | 0.0550 | 0.0800 | 0.0590 | 0.8439 | 0.8441 | 0.0210 | 0.0105 | 0.1040 | 0.1840 | 0.1748 | 0.5664 | 0.0154 |
| **S. Central** | 0.0240 | 0.0750 | 0.0910 | 0.0820 | 0.7310 | 0.7466 | 0.0210 | 0.0105 | 0.2440 | 0.3200 | 0.3354 | 0.6582 | 0.0154 |
| **Teso** | 0.0080 | 0.0140 | 0.0210 | 0.0270 | 0.8893 | 0.8964 | 0.0210 | 0.0105 | 0.0418 | 0.0400 | 0.0410 | 0.6393 | 0.0154 |
| **Tooro** | 0.0150 | 0.0370 | 0.0630 | 0.0720 | 0.8400 | 0.8507 | 0.0210 | 0.0105 | 0.1382 | 0.1520 | 0.1340 | 0.5052 | 0.0154 |
| **West Nile** | 0.0100 | 0.0410 | 0.0240 | 0.0350 | 0.8441 | 0.8578 | 0.0210 | 0.0105 | 0.1357 | 0.1010 | 0.0789 | 0.5921 | 0.0154 |
